# Proteins and carbohydrates in nipple aspirate fluid predict the presence of atypia and cancer in women requiring diagnostic breast biopsy

**DOI:** 10.1186/1471-2407-12-52

**Published:** 2012-02-01

**Authors:** Wenyi Qin, Gerald Gui, Ke Zhang, Dominique Twelves, Beth Kliethermes, Edward R Sauter

**Affiliations:** 1 Department of Surgery, University of North Dakota School of Medicine and Health Sciences, 501 N. Columbia Rd., Grand Forks, ND 58202, USA; 2 Department of Surgery and Pathology, University of North Dakota School of Medicine and Health Sciences, 501 N. Columbia Rd., Grand Forks, ND 58202, USA; 3 Royal Marsden NHS Foundation Trust, Fulham Road, London SW3 6JJ, UK; 4 University of North Dakota, 501 N. Columbia Rd, Rm 5092, Grand Forks, ND 58202, USA

**Keywords:** Breast cancer, Premalignant lesions, Biomarkers, Cancer prediction

## Abstract

**Background:**

Herein we present the results of two related investigations. The first study determined if concentrations in breast nipple discharge (ND) of two proteins (urinary plasminogen activator, uPA and its inhibitor, PAI-1) predicted the presence of breast atypia and cancer in pre- and/or postmenopausal women requiring surgery because of a suspicious breast lesion. The second study assessed if these proteins increased the predictive ability of a carbohydrate (Thomsen Friedenreich, TF) which we previously demonstrated predicted the presence of disease in postmenopausal women requiring surgery.

**Methods:**

In the first study we prospectively enrolled 79 participants from whom we collected ND, measured uPA and PAI-1 and correlated expression with pathologic findings. In the second study we analyzed 35 (uPA and PAI-1 in 24, uPA in an additional 11) ND samples collected from different participants requiring breast surgery, all of whom also had TF results.

**Results:**

uPA expression was higher in pre- and PAI-1 in postmenopausal women with 1) cancer (DCIS or invasive) vs. either no cancer (atypia or benign pathology, p = .018 and .025, respectively), or benign pathology (p = .017 and .033, respectively); and 2) abnormal (atypia or cancer) versus benign pathology (p = .018 and .052, respectively). High uPA and PAI-1 concentrations and age were independent predictors of disease in premenopausal women, with an area under the curve (AUC) of 83-87% when comparing diseased vs. benign pathology. uPA, TF, and age correctly classified 35 pre- and postmenopausal women as having disease or not 84-91% of the time, whereas combining uPA+PAI-1+TF correctly classified 24 women 97-100% of the time.

**Conclusions:**

uPA and PAI-1 concentrations in ND were higher in women with atypia and cancer compared to women with benign disease. Combining uPA, PAI-1 and TF in the assessment of women requiring diagnostic breast surgery maximized disease prediction. The assessment of these markers may prove useful in early breast cancer detection.

## Background

Cancer cell invasion and metastasis requires the degradation of the extracellular matrix (ECM) and basement membrane. This process is accomplished by several proteins, including those of the plasminogen activator (PA) system. Urokinase-type PA (uPA) plays a key role in ECM degradation. In women with breast cancer, uPA appears to promote cancer invasion and metastasis [1] through degradation of the ECM, stimulation of angiogenesis, alteration in cell migration and adhesion [2], and inhibition of apoptosis [3].

Plasminogen activator activity is inhibited by plasminogen activator inhibitor-1 (PAI-1) [4]. PAI-1 promotes breast cancer invasion and metastasis. Deficient PAI-1 expression in mice prevented local invasion and tumor vascularization of transplanted malignant keratinocytes. When PAI-1 expression was restored, invasion and associated angiogenesis were also restored, suggesting that host-produced PAI-1 is essential for cancer cell invasion and angiogenesis [5]. PAI-1 promotes angiogenesis by directly inhibiting proteases [6], suggesting that excessive plasmin proteolysis may prevent the assembly of tumor blood vessels. Possible mechanisms by which PAI-1 promotes breast cancer include prevention of excess ECM degradation, modulation of cell adhesion, a role in angiogenesis, and the stimulation of cell proliferation [1]. The association of uPA and PAI-1 expression with breast cancer is complex.

In a pooled analysis of 8377 breast cancer patients, higher uPA and PAI-1 levels in tumor tissue were related to worse prognosis [7]. The importance of the PA system in breast cancer detection was explored by us in a sample set ranging from healthy women to those with advanced breast cancer, demonstrating that both uPA and PAI-1 were useful in predicting which women had breast cancer [8]. Specifically, high levels in breast nipple aspirate fluid (NAF) of uPA and PAI-1 significantly contributed to a model which predicted which women had breast cancer. In that study, we observed that uPA and PAI-1 are concentrated in NAF (a type of nipple discharge-ND, in addition to spontaneous ND, which can be physiologic or pathologic) compared to plasma [8]. Pathologic spontaneous ND (PND) comes forth from one but not the other breast nipple and generally the breast with PND harbors a benign or malignant tumor [9]. On the other hand, physiologic spontaneous ND is generally from both breasts and is not associated with cancer. Both NAF and PND can be obtained non-invasively and contain concentrated secreted proteins, carbohydrates and lipids from the breast ductal epithelium, the cells that give rise to cancer. This fact, as well as the fact that ND is breast specific, undiluted by the contribution from other organs, suggests that it may be a better physiologic fluid than plasma to identify breast cancer biomarkers.

In this study we demonstrate in a specific population of participants (women with a suspicious breast lesion which required biopsy to exclude cancer) that the expression of uPA and PAI-1 is altered in women with breast atypia and cancer. Since these markers were more predictive of disease in pre- than in post-menopausal women, and were not perfectly accurate in predicting the presence of disease in either menopausal group, we wondered if additional marker(s), ideally one or more that was especially predictive of postmenopausal disease, would improve the ability of uPA and PAI-1 to predict the presence of breast atypia or cancer.

We previously reported [9] that the Thomsen-Friedenreich (TF; Galactose-β-(1 → 3)-N-acetyl-D-galactosamine) antigen, which is displayed on cell-surface proteins and lipids in 70% to 90% of adenocarcinomas of the breast [10], is upregulated in post- (but not pre-menopausal) women with breast atypia and cancer, correctly classifying either cancer or abnormal vs. benign pathology 83% of the time in postmenopausal women. Because uPA and PAI-1 was also predictive in postmenopausal women, we hoped that it might increase the predictive ability of TF in these women. Because uPA was more predictive in premenopausal women, we hoped that it would increase the predictive ability in this group. We therefore evaluated all three markers in a matched subset of the 124 samples in which TF analysis was reported [9] and sufficient ND remained.

## Methods

### Patients and samples

After receiving Institutional Review Board approval, we obtained informed consent, prospectively enrolled participants and collected 79 ND samples in Grand Forks, North Dakota, at the University of North Dakota, in Columbia, MO, at the Ellis Fischel Cancer Center and in London, UK, at the Royal Marsden Cancer Center. Participants were enrolled from January 2008 to February 2010. Details of TF sample collection and analysis were previously reported [9]. Among the 124 ND samples collected in the TF report there was sufficient remaining NAF to analyze both uPA and PAI-1 in 24 and uPA alone in an additional 11 samples. Criteria for enrollment were the same in the current as in the TF report. A subject was classified as postmenopausal if at least one year had passed without a menstrual period or she had undergone bilateral oophorectomy prior to enrollment. Women who had undergone hysterectomy without bilateral oophorectomy were considered postmenopausal if they were over 50 years old. If follicle stimulating hormone (FSH) levels were available, a level of 34 mIU/mL or greater was used to classify women as postmenopausal. All ND samples were collected prior to excisional biopsy or mastectomy. Comparisons of uPA, PAI-1 and TF were based on the histopathologic findings in the clinical report. For the purposes of this report, we define "cancer" as pathologic evidence of either ductal carcinoma *in situ *(DCIS) or invasive cancer; "no cancer" as histopathology which was normal, usual hyperplasia, and/or atypical hyperplasia; "benign" as histopathology containing normal findings and/or usual hyperplasia; and "abnormal" as histopatology demonstrating atypical hyperplasia, DCIS, and/or invasive cancer.

### Sample collection

ND (1-10 μL) was obtained from the breast with a lesion prior to surgery. Lesions included women with 1) pathologic nipple discharge (PND); 2) a suspicious lesion identified on imaging, be it mammogram, ultrasound or breast magnetic resonance imaging; and/or 3) a palpable lesion that was not a simple cyst. Samples were collected as described previously [11]. Briefly, after informed consent was obtained, ND fluid was aspirated using a breast pump (NAF) or collected after the participant massaged her breast (PND). Samples were collected into capillary tubes, the volume of NAF measured and stored at -80°C until use.

### Preparation

The portion of the capillary tube containing the sample was introduced into a 1.7 mL Eppendorf tube and 100 μL of a 0.1 mol/L solution of sodium bicarbonate (pH 7.8) added. The capillary tube was then crushed with a glass rod and the mixture vortexed to disperse the sample. The crushed capillary tube was left in the bicarbonate buffer overnight at 4°C, and the mixture then centrifuged (14,000 g, 5 min) and the supernatant used without further dilution.

### Analysis

*uPA and PAI-1*. ELISA kits for uPA and PAI-1 were obtained from American Diagnostica, Inc. (Greenwich, CT). Levels of these two markers in ND samples were determined according to the manufacturer's instructions. Briefly, 100 uL of standards, samples and blanks were pipetted into microplate wells coated with monoclonal antibodies respectively specific for uPA and PAI-1 and incubated overnight at 4°C. After washing (× 4), enzyme-linked antibodies specific for each analyte were added to the wells and incubated for 1 h at room temperature. The wells were washed again, diluted enzyme conjugate (streptavidin conjugated horseradish peroxidase) was pipetted into the wells, incubated (1 h, RT), then washed again. Substrate reagent was added to each well, followed by a stop solution (0.5 M sulfuric acid). Absorbance was measured with a microplate reader. Detection limits were 10 pg/mL for uPA and 50 pg/mL for PAI-1.

*TF*. The evaluation of this carbohydrate was previously reported [9].

### Statistical analysis

Biomarker levels for uPA and PAI-1 in the ND samples were heavily skewed and not normally distributed. We therefore described and analyzed biomarker levels using medians and log-transformed (Log10) means to achieve normal distributions. We performed a Shapiro-Wilk test to determine the goodness-of-fit of the data to a normal distribution. None of the log-transformed variables were significantly different from a normal distribution. Unpaired t-tests were calculated to determine significant differences between log10 means of the biomarkers. Logistic regression analyses are based on log10 transformed data. Three logistic regression models were used to examine the effects of uPA and PAI-1 in predicting (a) cancer vs. no cancer diagnosis; (b) cancer vs. benign diagnosis; and (c) abnormal vs. benign diagnosis. Various demographic factors, include age, menopausal status, and hormone replacement, were included in the logistic models as covariates. Because menopausal status was a near significant predictor in all three overall logistic models, follow-up logistic models were conducted separately for pre- and for post-menopausal women, controlling for age. Receiver operating characteristic (ROC) curves were calculated based on the logistic regression results for the premenopausal women's data, and area under the curve (AUC) values are presented. The optimal subset of all variants, uPA, PAI-1, TF, Tn, and covariates was selected to achieve the best AUC value.

## Results

### Recruitment

NAF was successfully collected in 90% (76/84) and PND in 100% (3/3) women. Among the 79 women evaluated for uPA and PAI-1 expression, 41 (51.9%) were postmenopausal. Age ranged from 21 to 82 years, with a median age in pre- and postmenopausal women of 44 and 61, respectively. Among the 35 women evaluated for TF, uPA +/- PAI-1 expression, 17 (48.6%) were postmenopausal. Age ranged from 26 to 77 years, with a median age in pre- and postmenopausal women of 43.5 and 61 years, respectively.

### Fluid volume and biomarker expression not different among women with vs. those without PND

ND volume ranged from 4 to 521 μl, with a mean of 60.9 and median of 29 μl. ND volume did not vary by pathologic diagnosis nor by fluid type (NAF vs. PND). We first determined if uPA and PAI-1 expression in women requiring diagnostic biopsy due to PND differed from expression in women requiring biopsy who did not have PND. Results of logistic regression analyses conducted using samples from women with PND and NAF showed that fluid type did not affect expression levels, with p > 0.5 for all biomarkers. Therefore, all reported analyses include both PND and NAF samples (N = 79).

### uPA concentration is associated with breast atypia and cancer

uPA concentration was higher (Table 1) in women with cancer (DCIS or invasive) than in women with 1) no cancer: atypia or benign pathology (p = .023), and 2) benign pathology (p = .025). uPA was also higher in women with abnormal (atypia or cancer) than benign pathology (p = .050). PAI-1 was higher in women with cancer (DCIS or invasive) than in women with benign pathology (p = .037).

**Table 1:** Univariate analyses of uPA and PAI-1 expression (ng/mL) in women requiring breast surgery

A. Cancer/no cancer		Overall	No cancer^1^	Cancer^1^	P value
***All Subjects (N)***		79	46	33	
uPA	Mean (SD)	2.4 (.90)	2.2 (.84)	2.7 (.92)	**.023**
	Median	2.4	2.2	2.6	
PAI-1	Mean (SD)	3.7 (1.5)	3.4 (1.4)	4.2 (1.6)	.064
	Median	3.5	3.3	4.1	
***Premenopausal (N)***		38	23	15	
uPA	Mean (SD)	2.5 (.93)	2.2 (.82)	2.9 (.93)	**.018**
	Median	2.5	2.3	2.7	
***Postmenopausal (N)***		41	23	18	
PAI-1	Mean (SD)	4.0 (1.6)	3.5 (1.6)	4.6 (1.4)	**.025**
	Median	3.8	3.3	4.3	
**B. Cancer/benign**		**Overall**	**Benign^1^**	**Cancer^1^**	**P value**
***All Subjects (N)***		72	39	33	
uPA	Mean (SD)	2.4 (.93)	2.2 (.89)	2.7 (.92)	**.025**
	Median	2.4	2.2	2.6	
PAI-1	Mean (SD)	3.7 (1.5)	3.4 (1.4)	4.2 (1.6)	**.037**
	Median	3.4	3.1	4.1	
***Premenopausal (N)***		37	22	15	
uPA	Mean (SD)	2.4 (.95)	2.2 (.84)	2.9 (.93)	**.017**
	Median	2.5	2.2	2.7	
***Postmenopausal (N)***		35	17	18	
PAI-1	Mean (SD)	4.0 (1.7)	3.4 (1.7)	4.6 (1.4)	**.033**
	Median	3.8	2.8	4.3	
**C. Abnormal/benign**		**Overall**	**Benign^1^**	**Abnormal^1^**	**P value**
***All Subjects (N)***		79	39	40	
uPA	Mean (SD)	2.4 (.90)	2.2 (.89)	2.6 (.88)	**.050**
	Median	2.4	2.2	2.5	
PAI-1	Mean (SD)	3.7 (1.5)	3.4 (1.4)	4.0 (1.6)	.060
	Median	3.5	3.1	3.9	
***Premenopausal (N)***		38	22	16	
uPA	Mean (SD)	2.5 (.93)	2.2 (.84)	2.9 (.91)	**.018**
	Median	2.5	2.2	2.7	
***Postmenopausal (N)***		41	17	24	
PAI-1	Mean (SD)	4.0 (1.6)	3.4 (1.7)	4.4 (1.3)	.052
	Median	3.8	2.8	4.1	

^1^No cancer = normal, hyperplasia, and atypical hyperplasia; Cancer = ductal carcinoma *in situ *and invasive cancer; Benign = normal and hyperplasia; Abnormal = atypical hyperplasia, ductal carcinoma *in situ *and invasive cancer. Means and medians reflect log10 values for each marker. For all subjects, all p values are provided regardless of the result. For pre- and postmenopausal groupings, only results which were significant or approached significance (p < .065) were included

### uPA and PAI-1 expression based on menopausal status

Univariate analysis indicated that uPA concentration was more predictive of disease in premenopausal women, and PAI-1 in postmenopausal women (Table 1). uPA concentration was higher in premenopausal women with cancer (DCIS or invasive) than in women with 1) no cancer (p = .018), and 2) benign pathology (p = .017), and in women with abnormal pathology than those with benign pathology (p = .018). PAI-1 concentration was higher in postmenopausal women with cancer (DCIS or invasive) than in women with 1) no cancer (p = .025), and 2) benign pathology (p = .033).

In 79 ND samples, we generated ROC curves to determine how well information on uPA and age predicted if a premenopausal woman had breast atypia or cancer. Three comparisons: cancer vs. no cancer, cancer vs. benign pathology, and abnormal vs. benign pathology were conducted. The AUC values ranged from .83-.87 (Table 2), and were better than those for postmenopausal women.

**Table 2:** AUC values for disease prediction considering uPA, PAI-1, TF and age^1^

**Biomarker**	**Group**	**N^1^**	AUC		
			**CA/no CA^2^**	**CA/benign^2^**	**Abnormal/benign^2^**
uPA+PAI-1	All	79	.72	.75	.74
uPA^3^	Pre	38	.87	.83	.86
TF^4^	Post	72	.81	.83	.83
TF+uPA	All	35	.84	.92	.91
TF+uPA+PAI-1	All	24	1.0	1.0	.97

^1^AUC: area under the receiver operating curve; uPA: urinary plasminogen activator; PAI (uPA inhibitor)-1, TF: Thomsen-Friedenreich antigen; N = number or sample size. The equivalent values as percent should be multiplied by 100 (i.e., .97 = 97%, and so forth)

^2^Cancer = ductal carcinoma *in situ *(DCIS), and invasive cancer; Benign = normal and hyperplasia; Abnormal = atypical hyperplasia, DCIS, and invasive cancer. Age is in all three models

^3^AUC values for postmenopausal women were lower

^4^previously published data [9]

### The combination of TF, uPA and PAI-1 are highly predictive of the presence of breast cancer

**Figure 1:**
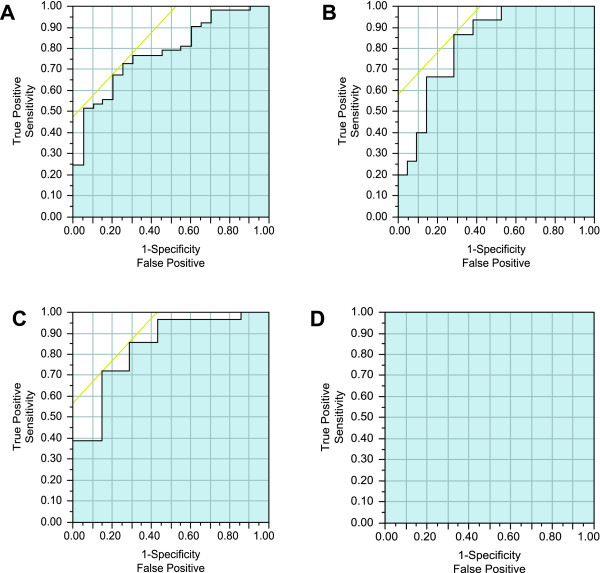
**ROC curves for the prediction of breast cancer**. TF, uPA, PAI-1 and age information were used to generate curves comparing cancer = DCIS and invasive cancer vs. no cancer = normal, hyperplasia and atypical hyperplasia: TF and age in postmenopausal women (AUC = .81) **(A)**, uPA and age in premenopausal women (AUC = .87) **(B)**; TF, uPA and age in all (pre- and postmenopausal) women (AUC = .84) **(C); **and TF, uPA, PAI-1 and age in all women (AUC = 1.0) **(D)**. AUC for each illustration is indicated by blue shading.

Among the 79 samples evaluated for uPA+PAI-1 expression, the AUC for age alone was .62. The AUC for uPA, PAI-1 and age ranged from .72 to .75 when predicting disease in all women, whereas the AUC for uPA and age in premenopausal women only was higher (.83-.87). We then measured uPA and PAI-1 in NAF collected contemporaneously with that used to measure TF. For all of these analyses, age was in the predictive model. TF+uPA predicted disease in both pre- and postmenopausal women with 84-92% accuracy. When TF, uPA, and PAI-1 were combined, the predictive ability approached 100% (Table 2, Figure 1).

## Discussion

Carbohydrate biomarkers have not been investigated as extensively as proteins in NAF or other bodily fluids. Nonetheless, there have been studies dating back to at least 1989 in which the carbohydrate lactose was measured in [12]. Lactose, whose presence was felt to indicate evidence of breast secretory activity, was detected in 44.8% of the samples overall, declining from being detected in 100% of samples from women less than age 30 to being detected in 29% of samples from women ≥ 35 years old. A more recent study demonstrated that dietary intake of lactose was a strong predictor of the ability to collect NAF [13]. The measurement of TF antigen in NAF from 23 women with biopsy proven breast cancer using the galactose oxidase-Schiff (GOS) reaction was reported in 2004 [14]. The change in color with the reaction was significantly different between NAF from cancer containing vs. NAF from healthy contralateral breasts. We reported [9] levels of TF measured by direct immunoassay in 124 ND samples, including 52 from pre- and 72 from post-menopausal women with a suspicious breast lesion which required biopsy. Age alone in this cohort provided an AUC of .69. TF and age predicted the presence of atypia and cancer in the postmenopausal group with an AUC of .83. In an analysis of 79 ND samples (different than the 124) with similar enrollment criteria, uPA was highly predictive of breast cancer in premenopausal women (.83-.87), but less so in postmenopausal women. We then determined that two markers were better than one in predicting disease in all women, with TF + uPA having an AUC of .84-.92. When TF, uPA, and PAI-1 were combined, the AUC approached 1.0%.

We previously found that the expression of some ND cancer prediction markers varied based on whether or not the subject requiring surgery did or did not have PND [15]. On the other hand, TF concentration was not influenced by the presence or absence of PND [9]. We therefore determined if uPA and PAI-1 concentrations differed based on whether the sample was PND or NAF. Since we did not identify a concentration difference, all analyses included both PND and NAF samples.

We have three NAF biomarkers (TF, uPA and PAI-1) which together appear highly predictive of the presence of cancer or precancer in women requiring breast biopsy to exclude disease. The markers appear to be highly predictive of disease, regardless of whether or not PND was present. The NAF markers appear complementary, as two of the biomarkers (TF and PAI-1) are more predictive in post- than premenopausal women, whereas uPA is more predictive in premenopausal women.

The study had limitations. The first limitation of the study was the small sample size for the analysis of all three markers. Second, TF was analyzed at a different time than uPA and PAI-1. How much the age of the samples influenced marker expression is uncertain, though we did determine that uPA and PAI-1 expression was similar between the groups of 79 and 24 samples. A validation study in a larger sample size would help to determine the predictive ability of uPA, PAI-1 and TF would help to address these limitations.

## Conclusions

In women requiring breast biopsy to exclude disease, combining ND biomarkers that were each predictive of the presence of atypia or cancer improved the prediction of both atypia and cancer, regardless of whether or not PND was present. A validation study is warranted to determine the clinical use of these noninvasive markers.

## Abbreviations

Abs: Absorbance; AH: Atypical hyperplasia; AUC: Area under the curve; CV: Coefficient of variation; DCIS: Ductal carcinoma *in situ*; H: Usual hyperplasia; ICC: Intraclass correlation coefficient; NAF: Nipple aspirate fluid; ND: Nipple discharge; OR: Odds ratio; PND: Pathologic nipple discharge; ROC: Receiver operating characteristic.

## Competing interests

Dr. Sauter is a consultant for Atossa Genetics, Inc.

## Authors' contributions

Dr. Qin performed specimen analysis. Drs. Gui and Twelves enrolled participants and critically reviewed the manuscript. Dr. Zhang performed the statistical analysis. Ms. Kliethermes performed data entry, managed the database and revised the manuscript. Dr. Sauter designed and oversaw all aspects of the study, enrolled participants, and prepared the preliminary and final drafts of the manuscript. All authors read and approved the final manuscript.

## Pre-publication history

The pre-publication history for this paper can be accessed here:

http://www.biomedcentral.com/1471-2407/12/52/prepub

## References

[B1] Duffy MJ Urokinase plasminogen activator and its inhibitor, PAI-1, as prognostic markers in breast cancer: from pilot to level 1 evidence studies Clin Chem 2002 48 8 1194 1197 12142372

[B2] Andreasen PA Kjoller L Christensen L Duffy MJ The urokinase-type plasminogen activator system in cancer metastasis: a review Int J Cancer 1997 72 1 1 22 10.1002/(SICI)1097-0215(19970703)72:1<1::AID-IJC1>3.0.CO;2-Z 9212216

[B3] Ma Z Webb DJ Jo M Gonias SL Endogenously produced urokinase-type plasminogen activator is a major determinant of the basal level of activated ERK/MAP kinase and prevents apoptosis in MDA-MB-231 breast cancer cells J Cell Sci 2001 114 Pt 18 3387 3396 11591826 10.1242/jcs.114.18.3387

[B4] Blasi F Proteolysis, cell adhesion, chemotaxis, and invasiveness are regulated by the u-PA-u-PAR-PAI-1 system Thromb Haemost 1999 82 2 298 304 10605717

[B5] Bajou K Noel A Gerard RD Masson V Brunner N Holst-Hansen C Skobe M Fusenig NE Carmeliet P Collen D Absence of host plasminogen activator inhibitor 1 prevents cancer invasion and vascularization Nat Med 1998 4 8 923 928 10.1038/nm0898-923 9701244

[B6] Bajou K Masson V Gerard RD Schmitt PM Albert V Praus M Lund LR Frandsen TL Brunner N Dano K The plasminogen activator inhibitor PAI-1 controls in vivo tumor vascularization by interaction with proteases, not vitronectin. Implications for antiangiogenic strategies J Cell Biol 2001 152 4 777 784 10.1083/jcb.152.4.777 11266468 PMC2195770

[B7] Look MP van Putten WL Duffy MJ Harbeck N Christensen IJ Thomssen C Kates R Spyratos F Ferno M Eppenberger-Castori S Pooled analysis of prognostic impact of urokinase-type plasminogen activator and its inhibitor PAI-1 in 8377 breast cancer patients J Natl Cancer Inst 2002 94 2 116 128 10.1093/jnci/94.2.116 11792750

[B8] Qin W Zhu W Wagner-Mann C Folk W Sauter ER Association of uPA, PAT-1, and uPAR in nipple aspirate fluid (NAF) with breast cancer Cancer J 2003 9 4 293 301 10.1097/00130404-200307000-00012 12967140

[B9] Deutscher SL Dickerson M Gui G Newton J Holm JE Vogeltanz-Holm N Kliethermes B Hewett JE Kumar SR Quinn TP Carbohydrate antigens in nipple aspirate fluid predict the presence of atypia and cancer in women requiring diagnostic breast biopsy BMC Cancer 2010 10 1 519 10.1186/1471-2407-10-519 20920311 PMC2958935

[B10] Springer GF T and Tn, general carcinoma autoantigens Science 1984 224 4654 1198 1206 10.1126/science.6729450 6729450

[B11] Sauter ER Ross E Daly M Klein-Szanto A Engstrom PF Sorling A Malick J Ehya H Nipple aspirate fluid: a promising non-invasive method to identify cellular markers of breast cancer risk Br J Cancer 1997 76 4 494 501 10.1038/bjc.1997.415 9275027 PMC2228000

[B12] Petrakis NL Lim ML Miike R Lee RE Morris M Lee L Mason L Nipple aspirate fluids in adult nonlactating women-lactose content, cationic Na+, K+, Na+/K+ ratio, and coloration Breast Cancer Res Treat 1989 13 1 71 78 10.1007/BF01806552 2706328

[B13] Huang Y Anderson KE Nagamani M Grady JJ Lu LJ Dietary intake of lactose as a strong predictor for secretor status of nipple aspirate fluid in healthy premenopausal nonlactating women Clin Cancer Res 2008 14 5 1386 1392 10.1158/1078-0432.CCR-07-4077 18316559 PMC2690957

[B14] Chagpar A Evelegh M Fritsche HA Jr Krishnamurthy S Hunt KK Kuerer HM Prospective evaluation of a novel approach for the use of a quantitative galactose oxidase-Schiff reaction in ductal fluid samples from women with breast carcinoma Cancer 2004 100 12 2549 2554 10.1002/cncr.20311 15197795

[B15] Sauter ER Wagner-Mann C Ehya H Klein-Szanto A Biologic markers of breast cancer in nipple aspirate fluid and nipple discharge are associated with clinical findings Cancer Detect Prev 2007 31 1 50 58 10.1016/j.cdp.2006.12.004 17317033 PMC1865519

